# Quality of Life and Its Correlates in Alcohol Use Disorder Patients With and Without Depression in China

**DOI:** 10.3389/fpsyt.2020.627338

**Published:** 2021-01-22

**Authors:** Hui Huang, Hongxian Shen, Kui Ning, Ruiling Zhang, Wei Sun, Bing Li, Haifeng Jiang, Wenzheng Wang, Jiang Du, Min Zhao, Zhihua Yi, Jing Li, Rongxin Zhu, Shuiping Lu, Shiping Xie, Xiaoping Wang, Wei Fu, Chengge Gao, Wei Hao

**Affiliations:** ^1^Affiliated Wuhan Mental Health Center, Tongji Medical College, Huazhong University of Science and Technology, Wuhan, China; ^2^CAS Key Laboratory of Mental Health, Institute of Psychology, Chinese Academy of Sciences, Beijing, China; ^3^Key Laboratory of Psychiatry and Mental Health of Hunan Province, The China National Clinical Research Center for Mental Health Disorders, National Technology Institute of Psychiatry, Mental Health Institute of the Second Xiangya Hospital, Central South University, Changsha, China; ^4^Henan Mental Hospital, Xinxiang, China; ^5^Peking University Sixth Hospital, Beijing, China; ^6^Shanghai Mental Health Center, Shanghai, China; ^7^West China Hospital, Sichuan University, Chengdu, China; ^8^Nanjing Brain Hospital, Nanjing, China; ^9^Hubei General Hospital, Wuhan, China; ^10^The First Affiliated Hospital of Xian Jiaotong University, Xian, China

**Keywords:** alcohol use disorder, depression, quality of life, BDI, SF-36

## Abstract

**Objective:** Alcohol use disorder (AUD) is a serious issue worldwide and frequently co-occurs with depression. However, the quality of life (QOL) of AUD patients with and without depression is not well studied in the Chinese Han population. The aim of this study was to investigate QOL and its correlates in AUD patients with and without depression in China.

**Methods:** Five hundred and fifteen psychiatric patients diagnosed with AUD were recruited. All these patients completed the Beck Depression Inventory (BDI) to assess depression, the Medical Outcome Study 36-Item Short Form Health Survey (SF-36) to evaluate QOL and the Alcohol Use Disorders Identification Test (AUDIT) to measure the severity of drinking.

**Results:** Compared with AUD patients without depression, those with depression had a lower QOL in all eight domains of the SF-36 (all *P* < 0.001), but were more willing to have alcohol-related treatment (*P* < 0.05). Negative correlations were noted between (i) the BDI total score and all eight domains of the SF-36 (all *P* < 0.001); and (ii) between the AUDIT total score and six domains of the SF-36 (all *P* < 0.05).

**Conclusions:** Depression impairs QOL in patients with AUD in China. Early intervention in comorbid depression to improve QOL is needed.

## Introduction

Alcohol use disorder (AUD) is a severe public health and medical issue in China. According to the latest survey, lifetime AUD in China is among the top three most prevalent classes of mental disorders, at 4.4% for adults, following anxiety disorders (7.6%) and mood disorders (7.4%) (1). Although AUD has caused enormous medical, social and economic burdens and costs in China, the treatment rate for AUD is dramatically low (2). Considering the high prevalence of AUD, its effect on quality of life (QOL) is important and needs to be investigated.

QOL is a critical parameter that reflects the impact of a disease on the individual's well-being and includes physical, mental and social dimensions. The QOL of patients with AUD has received considerable attention. Accumulated research evidence has shown that AUD impairs QOL and causes faculty loss. Many studies found that AUD patients reported poorer QOL compared with the general population (3–7), particularly in the emotional (8–12), mental health (13, 14) and social functioning domains (11). However, the scores in the physical functioning domain were not significantly different between the AUD patients and the reference population (13, 14). Moreover, studies have also revealed that AUD is a risk factor (15, 16) or even a primary cause of low QOL (17), which means that AUD might be used to predict subsequent QOL. Moreover, evidence indicated that the higher alcohol consumption or longer heavy drinking days, the lower levels of QOL (5, 18, 19). Importantly, studies have reported that alcohol abstinence and its maintenance could improve QOL in AUD patients (8, 20–23).

The relationship between QOL deterioration and AUD was moderated by many factors, such as comorbidity with psychiatric disorders (4, 21). Previous studies reported a significant association between depression and diminished QOL in AUD patients (17, 24–26). For example, both Lee et al. and Saatcioglu et al. reported that AUD patients with depressive symptoms had poorer QOL compared with those without depressive symptoms (24, 25). According to the study from Ponizovsky et al., depression was associated with impaired QOL in alcohol-dependent patients (26). Moreover, Levola et al. conducted a review and found that depression considerably impacted QOL among patients with alcohol dependence (17). Interestingly, after treatment with escitalopram or memantine, significant improvement in QOL of the social functioning domain in alcohol-dependent patients with comorbid depression was observed (27).

Although AUD is a serious issue in China, the effect of depression on the QOL of AUD patients has not been studied thoroughly. Furthermore, previous studies regarding the impact of depression on QOL in AUD patients were performed in Western or Asian countries except for China (13, 24, 25, 28). To date, no study has assessed QOL in AUD patients with and without depression in China. Thus, we conducted the present hospital-based study to compare the QOL between AUD patients with and without depression and its correlates in the Chinese Han population. Our study will provide a reference for early intervention in QOL deterioration in AUD patients with depression.

## Methods

### Samples

The inclusion criteria for the study were patients aged ≥18 years who met the DSM-IV criterion for alcohol dependence or alcohol abuse. Potential participants were interviewed by trained and experienced psychiatrists to assess the inclusion criteria. A total of 546 patients were recruited from consecutive admissions to general psychiatric clinics in China. Thirty-one patients were excluded because they could not complete the entire interview. Ultimately, 515 patients were enrolled and completed the self-developed questionnaire, the Beck Depression Inventory (BDI) for depressive symptoms, the Medical Outcome Study 36-Item Short Form Health Survey (SF-36) for quality of life, and the Alcohol Use Disorders Identification Test (AUDIT) for the severity of drinking. Patients were allocated into two groups according to their BDI total scores: patients with no depression (BDI score ≤ 13, see below) and patients with depression(BDI score ≥14, see below). The study was approved by the Institutional Review Board of Second Xiangya Hospital of Central South University and informed consent for participating in the study was obtained from all patients.

### Instruments

We used a self-developed questionnaire to assess 515 patients for demographic characteristics, drinking patterns, comorbid disorders, drinking-related treatments and patients' attitudes and expectations for treatment. The validated Chinese version of BDI consists of 21 items and was utilized to assess depressive symptoms. The total score of the BDI ranges from 0 to 63. A cutoff point of 13 on the BDI total score was used to distinguish between AUD patients with (BDI score ≥14) and without (BDI score ≤ 13) depression (29). The validated Chinese version of the SF-36 was used to measure QOL (30). The SF-36 is a multidimensional instrument with 36 items that address eight QOL domains, including physical function, role physical, body pain, general health, vitality, social function, role emotional, and mental health. According to previous studies, each domain score was normalized to obtain a value between 0 and 100 inclusive [100^*^(score obtained - minimum score possible)/(maximum score possible - minimum score possible)], and a high score indicated a good QOL (5, 8). The AUDIT-Chinese version with 10 items was used to estimate the severity of drinking. Each item has a score ranging from 0 to 4. Thus, the maximum score for the total instrument is 40 points, and the minimum score is zero (31). All of the SF-36, AUDIT and BDI scales were subject to translation procedures, cultural adaptations and psychometric properties. No significant difference was found between the Chinese version and the original English version. Hence, these scales were suitable to the Chinese cultural context (30–32). All patients were diagnosed with alcohol dependence or alcohol abuse based on the Diagnostic and Statistical Manual of Mental Disorders-Fourth Edition (DSM-IV) (33). We merged alcohol dependence and abuse and labeled it as “alcohol use disorder (AUD)” in our study. The self-developed questionnaire, the BDI, the SF-36, the AUDIT, and the structured interview on AUD were administered by trained and experienced psychiatrists.

### Statistical Analysis

All statistical analyses were performed by SPSS version 20.0. Continuous data were described as the mean ± standard deviation, and categorical data were described as frequencies and percentages. The Kolmogorov-Smirnov one-sample test was used to measure the normality of distribution. Comparisons between the depression and non-depression AUD patients were made using Student's *t-*test (continuous variables), chi-squared test (categorical variables) and Mann-Whitney U test (nonnormally distributed data). In Mann-Whitney U test, Standardized Test Statistic (Z value) was reported. Pearson correlations were used to examine the relationship between QOL and clinical data. A value of *P* < 0.05 was considered significant. Bonferroni correction was used for the multiple comparison correction.

## Results

### Demographic Characteristics, Drinking Patterns and Comorbid Disorders of AUD Patients With and Without Depression

A total of 254 (49.3%) of 515 AUD patients met the criteria for depression, and 261 (50.7%) did not. The demographic characteristics and drinking patterns of the two groups were presented in Table 1. Compared with the non-depression group, the depression group had more females, was younger, and had more unstable marital status (all *P* < 0.05). No significant differences in other variables, including years of education, employment status, monthly family income, age at first drink, daily consumption of pure alcohol, drinking frequency, types of alcoholic beverages, and drinking-related treatments, were found (all *P* > 0.05). Comorbid disorders of AUD patients with and without depression were presented in Table 2.

**Table 1:** Demographic characteristics and drinking patterns of AUD patients with and without depression.

**Characteristics**	**Depression (*n* = 254)**	**Non-depression (*n* = 261)**	***t*/χ^2^/Z**	***P***
Gender			8.122	0.04
Males	228 (89.8%)	251 (96.2%)		
Females	26 (10.2%)	10 (3.8%)		
Age (years)	41.0 ± 10.3	43.0 ± 10.5	2.242	0.025
Education (years)	11.0 ± 3.5	11.2 ± 3.7	0.537	0.591
Marital status			12.297	0.006
Married/cohabiting	185 (72.8%)	222 (85.1%)		
Single	39 (15.4%)	24 (9.2%)		
Divorced/separated	29 (11.4%)	15 (5.7%)		
Widowed	1 (0.4%)	0 (0.0%)		
Employment status			2.267	0.132
Employed	184 (72.4%)	204 (78.2%)		
Unemployed	70 (27.6%)	57 (21.8%)		
Monthly family income(US$)			1.928	0.381
< $423	111 (44.0%)	128 (49.6%)		
$423–705	103 (40.9%)	99 (38.4%)		
>$705	38 (15.1%)	31 (12.0%)		
Age at first drink	18.4 ± 5.0	18.3 ± 4.9	0.119	0.905
Daily consumption of pure alcohol(g)	110.9 ± 102.5	100.0 ± 83.2	1.327	0.185
Drinking frequency			7.584	0.108
≥1/day	152 (59.8%)	147 (56.3%)		
4–6/week	43 (16.9%)	53 (20.3%)		
2–3/week	33 (13.0%)	47 (18.0%)		
2–4/month	25 (9.8%)	14 (5.4%)		
≤ 1/month	1 (0.4%)	0 (0.0%)		
Types of alcoholic beverages			0.524	0.769
Spirits	208 (81.9%)	217 (83.1%)		
Beer	37 (14.6%)	33 (12.6%)		
Others	9 (3.5%)	11 (4.2%)		
Drinking-related treatments			3.401	0.065
Present	64 (25.2%)	85 (32.6%)		
Absent	190 (74.8%)	176 (67.4%)		

**Table 2:** Comorbid disorders of AUD patients with and without depression.

**Comorbid disorders**	**Depression (*n* = 254)**	**Non-depression (*n* = 261)**	**χ^2^**	***P***
Schizophrenia	8 (3.1%)	14 (5.4%)	1.544	0.214
Bipolar disorder	12 (4.7%)	6 (2.3%)	2.245	0.134
Major depressive disorder	47 (18.5%)	4 (1.5%)	41.555	<0.001
Anxiety disorder	15 (5.9%)	17 (6.5%)	0.082	0.775
Drug use disorder	6 (2.4%)	3 (1.1%)	1.103	0.294
Others	13 (5.1%)	16 (6.1%)	0.248	0.618
Total	101 (39.8%)	60 (23.0%)	16.857	<0.001

### QOL and Its Correlation of AUD Patients With and Without Depression

Table 3 shows that AUD patients with depression had a lower QOL than those without depression in all domains. Significant differences in the physical functioning, role physical, role emotional, bodily pain, general health, vitality, social functioning, and mental health domains were noted (all *P* < 0.001). The Pearson correlation suggested that the BDI total score and the AUDIT total score was negatively correlated with each domain of the SF-36 (Table 4). After Bonferroni correction (0.05/16 = 0.003125), the BDI total score was still negatively correlated with each domain of the SF-36. Except for the bodily pain and mental health domain, all other domains were still negatively correlated with the AUDIT total score.

**Table 3:** Quality of life of AUD patients with and without depression.

**Domains of the SF-36**	**Depression**	**Non-depression**	**Z**	***P***
Physical functioning	80.5 ± 21.4	88.4 ± 15.5	4.579	<0.001
Role physical	49.8 ± 28.9	71.3 ± 25.1	8.169	<0.001
Role emotional	41.5 ± 25.2	71.0 ± 22.2	11.909	<0.001
Bodily Pain	64.7 ± 27.2	79.5 ± 21.8	6.181	<0.001
General health	31.9 ± 19.7	50.6 ± 23.1	8.889	<0.001
Vitality	37.3 ± 20.7	60.3 ± 18.7	11.305	<0.001
Social functioning	44.0 ± 23.1	68.9 ± 23.2	10.746	<0.001
Mental health	41.8 ± 17.6	69.6 ± 16.5	14.564	<0.001

**Table 4:** Correlations between the SF-36 and the BDI and AUDIT total score.

**Domains of the SF-36**	**BDI total score(r, *P*)**	**AUDIT total score(r, *P*)**
Physical functioning	−0.23, <0.001	−0.25, <0.001
Role physical	−0.41, <0.001	−0.27, <0.001
Role emotional	−0.59, <0.001	−0.20, <0.001
Bodily Pain	−0.38, <0.001	−0.10, 0.016^*^
General health	−0.44, <0.001	−0.23, <0.001
Vitality	−0.61, <0.001	−0.17, <0.001
Social functioning	−0.56, <0.001	−0.22, <0.001
Mental health	−0.71, <0.001	−0.08, 0.047^*^

* *Not statistically significant after Bonferroni adjustment*.

### Patients' Attitudes and Expectations for Treatment in AUD Patients With and Without Depression

One hundred and forty-nine patients had been treated for alcohol intoxication or alcohol withdrawal or alcohol-related liver injury. Pharmacotherapy primarily included vitamin B_1_ (84.5%), benzodiazepines (79.8%), hepatinica (65.1%) and naloxone (10.7%). No significant difference in drinking-related treatments was found between AUD patients with and without depression(χ^2^ = 3.401, *P* = 0.065). Two hundred and twenty-two (87.4%) depressed AUD patients thought they needed treatment, whereas the rate was 76.9% in non-depressed AUD patients. The difference was significant (χ^2^ = 9.600, *P* = 0.002). Expectations for the treatment of depressed AUD patients were alcohol abstinence (70.4%), alcohol reduction (27.3%) and others (2.3%); these rates in non-depressed AUD patients were 59.8, 39.2, and 1.0%, respectively. The difference between these two groups was significant (χ^2^ = 7.102, *P* = 0.029).

## Discussion

To the best of our knowledge, the present study is the first hospital-based survey to investigate QOL and its correlates in AUD patients with and without depression in the Chinese Han population. We found that depressed AUD patients had a lower QOL in all eight domains of the SF-36 and were more willing to take alcohol-related treatment than non-depressed AUD patients. We also observed that the BDI total score were negatively correlated with all eight domains of the SF-36 and the AUDIT total score were negatively correlated with six domains of the SF-36.

We found that AUD patients comorbid with depression had a lower QOL compared to those without depression and that the level of QOL negatively correlated with the severity of depression, suggesting that depression might be a significant predictor of poor QOL. Our main findings were consistent with previous studies in other Asian countries (24–26). For example, two studies conducted in South Korea reported that the score of QOL negatively correlated with the BDI score in patients with alcohol dependence (34) and that depression could impair QOL in AUD patients (24). Moreover, surveys from Turkey and Israel also revealed that depressive symptoms were significantly associated with QOL in alcohol-dependent patients (25, 26). Despite of socio-cultural and economic differences, similar results have also been observed in European and American countries (13, 14, 19, 35). For example, Malet et al. found that comorbid with depressive disorder was correlated with diminished QOL among patients diagnosed with alcohol dependence in France (13). According to the study from Daeppen et al., alcohol-dependent individuals in Switzerland with comorbid depressive symptoms was 21–127% lower than that among patients without comorbid depressive symptoms, especially in the psychosocial domain (14). Furthermore, Rosenbloom et al. found that depressive symptoms were associated with decreased QOL in American patients with alcohol dependence and that QOL was lower in individuals who had recovered from depression compared with those who had not (28). Importantly, Levola et al. conducted a systematic review and summarized that depression deteriorated the QOL of these patients (17). Taken together, studies in different countries around the world have consistently found that depression diminishes the QOL of AUD patients, suggesting the urgent need to provide interventions for comorbid depression.

Although many studies have reported that depression diminishes QOL in AUD patients, the mechanism of this phenomenon remains unclear. One study showed that alcohol-dependent patients comorbid with depression had poorer QOL in the social functioning domain compared with patients without depression due to the lack of socioeconomic resources and social support (24). Furthermore, self-confidence and self-esteem were significantly associated with QOL (26); alcohol-dependent patients with depression had much lower self-confidence and self-esteem than patients without depression, contributing to lower QOL (35). Importantly, depression itself deteriorates QOL. Major depression exhibited significant adverse effects on QOL in all domains: psychological functioning, physical functioning, social functioning, and role functioning (36). In addition, major depressive disorders accounted for 55% of QOL loss at the population level (37). Because QOL was impaired in AUD patients with depression, depression probably triggered alcohol relapse (25). Interestingly, we were surprised to find that compared with AUD patients without depression, those with depression were more willing to take alcohol-related treatment. This result is consistent with one previous study which reported that patients who were depressed were more likely to seek treatment (38).

The recovery goal for AUD has traditionally been alcohol abstinence. Nevertheless, abstinence might discourage patients from seeking treatment and does not generate changes in other fields of life (17). The improvement in QOL has gradually become a major indicator of recovery from AUD and is recognized as an ultimate treatment goal (25) and assessment of QOL would help to identify treatment demand in alcohol-dependent patients (39). Studies showed that reducing alcohol consumption or receiving treatment initiation could improve QOL in AUD patients (8, 19–23), especially in the mental health (9, 11, 40) and social functioning (11, 41) dimensions. For instance, according to the studies from Daeppen et al. and Dawson et al., the mental health domain of QOL was significantly improved after full or partial remission among alcohol-dependent patients (9, 40). Furthermore, Muhonen et al. conducted a double-blind randomized controlled trial among alcohol-dependent outpatients with major depressive disorder and found that the domain of social functioning was improved after treatment with escitalopram or memantine (27). Taken together, QOL may be used as an important outcome indicator of treatment and recovery from AUD.

Several limitations of our study should be mentioned. First, given the cross-sectional design of the study, it was unable to ascertain causality between comorbid with depression and impaired QOL in AUD patients. Second, the depression and non-depression group were not matched, especially for gender and age. Third, although the severity of depression was measured with the BDI, the diagnosis of depression was not established based on the DSM-IV. Fourth, information about family history and psychotropic drug treatment, which were potential factors associated with QOL, was not collected.

In conclusion, depression impairs QOL in all eight domains among patients with AUD in the Han Chinese population. Moreover, QOL negatively correlates with the BDI total score and the AUDIT total score. Early detection and intervention for comorbid depression and its correlates to improve QOL are needed.

## Data Availability Statement

The raw data supporting the conclusions of this article will be made available by the authors, without undue reservation.

## Ethics Statement

The studies involving human participants were reviewed and approved by the Institutional Review Board of Second Xiangya Hospital of Central South University. The patients/participants provided their written informed consent to participate in this study.

## Author Contributions

WH designed the study. HH, HS, KN, RZha, WS, BL, HJ, WW, JD, MZ, ZY, JL, RZhu, SL, SX, XW, WF, and CG collected the sample and performed the literature review. HH conducted the analyses and wrote the initial version of this manuscript. All authors edited, read, and approved the last version of this manuscript.

## Conflict of Interest

The authors declare that the research was conducted in the absence of any commercial or financial relationships that could be construed as a potential conflict of interest.
